# A review on deep learning applications in highly multiplexed tissue imaging data analysis

**DOI:** 10.3389/fbinf.2023.1159381

**Published:** 2023-07-26

**Authors:** Mohammed Zidane, Ahmad Makky, Matthias Bruhns, Alexander Rochwarger, Sepideh Babaei, Manfred Claassen, Christian M. Schürch

**Affiliations:** ^1^ Department of Pathology and Neuropathology, University Hospital and Comprehensive Cancer Center Tübingen, Tübingen, Germany; ^2^ Department of Internal Medicine I, University Hospital Tübingen, Tübingen, Germany; ^3^ Department of Computer Science, University of Tübingen, Tübingen, Germany

**Keywords:** artificial intelligence, deep learning, highly multiplexed tissue imaging, spatial transcriptomics, cancer, biomarker, prediction, review

## Abstract

Since its introduction into the field of oncology, deep learning (DL) has impacted clinical discoveries and biomarker predictions. DL-driven discoveries and predictions in oncology are based on a variety of biological data such as genomics, proteomics, and imaging data. DL-based computational frameworks can predict genetic variant effects on gene expression, as well as protein structures based on amino acid sequences. Furthermore, DL algorithms can capture valuable mechanistic biological information from several spatial “omics” technologies, such as spatial transcriptomics and spatial proteomics. Here, we review the impact that the combination of artificial intelligence (AI) with spatial omics technologies has had on oncology, focusing on DL and its applications in biomedical image analysis, encompassing cell segmentation, cell phenotype identification, cancer prognostication, and therapy prediction. We highlight the advantages of using highly multiplexed images (spatial proteomics data) compared to single-stained, conventional histopathological (“simple”) images, as the former can provide deep mechanistic insights that cannot be obtained by the latter, even with the aid of explainable AI. Furthermore, we provide the reader with the advantages/disadvantages of DL-based pipelines used in preprocessing highly multiplexed images (cell segmentation, cell type annotation). Therefore, this review also guides the reader to choose the DL-based pipeline that best fits their data. In conclusion, DL continues to be established as an essential tool in discovering novel biological mechanisms when combined with technologies such as highly multiplexed tissue imaging data. In balance with conventional medical data, its role in clinical routine will become more important, supporting diagnosis and prognosis in oncology, enhancing clinical decision-making, and improving the quality of care for patients.

## Introduction

Cancer is one of the main causes of morbidity and mortality worldwide (Siegel et al., 2023). Time to diagnosis, tumor grading, and staging all have a significant impact on how successfully cancer is treated (Gill et al., 2004; Cone et al., 2020). The development of advanced optical imaging technologies has significantly enhanced nondestructive single-cell analysis, offering the benefits of high sensitivity and low cost (Sun et al., 2020). Single-cell imaging aids in understanding of bodily functions and health, including treatment determination, tumor identification, and the analysis of metabolism (Veelken et al., 2017). Pathologists and researchers have employed conventional tissue microscopy techniques, such as immunohistochemistry and immunofluorescence, for decades to examine different cell types, cell abundances, and cell-cell interactions, determining cellular and subcellular protein structures. Nevertheless, in most cases, these techniques examine only a small range of relevant markers in tissue sections, with the full spectrum of intricacies hardly depicted adequately (Maric et al., 2021). Various highly multiplexed tissue imaging (HMTI) techniques were developed to overcome these limitations, and numerous deep learning (DL) methods were subsequently developed to analyze the highly multiplexed images. HMTI methods and DL applications in biology and medicine were recently reviewed elsewhere (Min et al., 2017; Suzuki, 2017; Tan WCC. et al., 2020; Echle et al., 2021; Einhaus et al., 2023). In this review, we will cover the novel space of DL applications in HMTI data.

### Highly multiplexed tissue imaging (HMTI)

Studies in biology, consortia research, and clinical medicine currently use multiplexed imaging methods to focus on spatial and structural correlations (Goltsev et al., 2017; Snyder et al., 2019; Schürch et al., 2020). The number of distinguishable cell types in tissue has been elevated with the recent development of HMTI technologies that enable imaging of samples with more than 40 markers simultaneously (Bodenmiller, 2016). As a result, multiplexed methods surpass the limitations of conventional immunophenotyping techniques by allowing for the spatial analysis of both phenotypically and functionally defined cell types. The simultaneous study of millions of cells with dozens of markers enables better comprehension of both disease and the tremendous complexity of organs (Chevrier et al., 2018). Consequently, HMTI produces large volumes of data, with treatment strategies becoming more specific as changes in the tissue environment, cell phenotypes, and neighborhood interactions are taken into account (Jarosch et al., 2021).

Numerous multiplexed tissue imaging techniques have been created over the last decade (Black et al., 2021). As a large fraction of HMTI techniques incorporate antibody staining, they can be grouped into two main categories based on antibody detection. The first category includes mass spectrometry-based imaging that enables imaging with more than 40 markers, including Imaging Mass Cytometry (IMC) (Giesen et al., 2014) and Multiplexed Ion Beam Imaging (MIBI) (Angelo et al., 2014). Both techniques depend on metal isotope-labeled antibodies, with the difference being the mode of ionization.

The second category of HMTI techniques are cyclic imaging methods in which staining is performed with either fluorophore- or DNA-tagged antibodies. The first of these approaches is based on cyclic *in situ* staining with fluorescent antibodies, image acquisition, and fluorescence elimination (Schubert et al., 2006). Examples of such approaches are termed multiplexed fluorescence microscopy (MxIF) (Gerdes et al., 2013) and multiepitope-ligand cartography (MELC) (Schubert et al., 2006). Removing the fluorescence by bleaching is what distinguishes MELC from MxIF, in which fluorescence is inactivated chemically. The other approach of cyclic imaging methods is the application of DNA-conjugated antibodies. Detection of such antibodies is performed by cyclic attachment and removal of complementary fluorophore-tagged DNA probes (Kennedy-Darling et al., 2020), as in the latest version of CO-Detection by indEXing (CODEX) (Goltsev et al., 2017). In Immunostaining with Signal Amplification By Exchange Reaction (ImmunoSABER), antigen detection events are amplified by expansion of repetitive binding sites for the complementary DNA probes (Saka et al., 2019).

**FIGURE 1:**
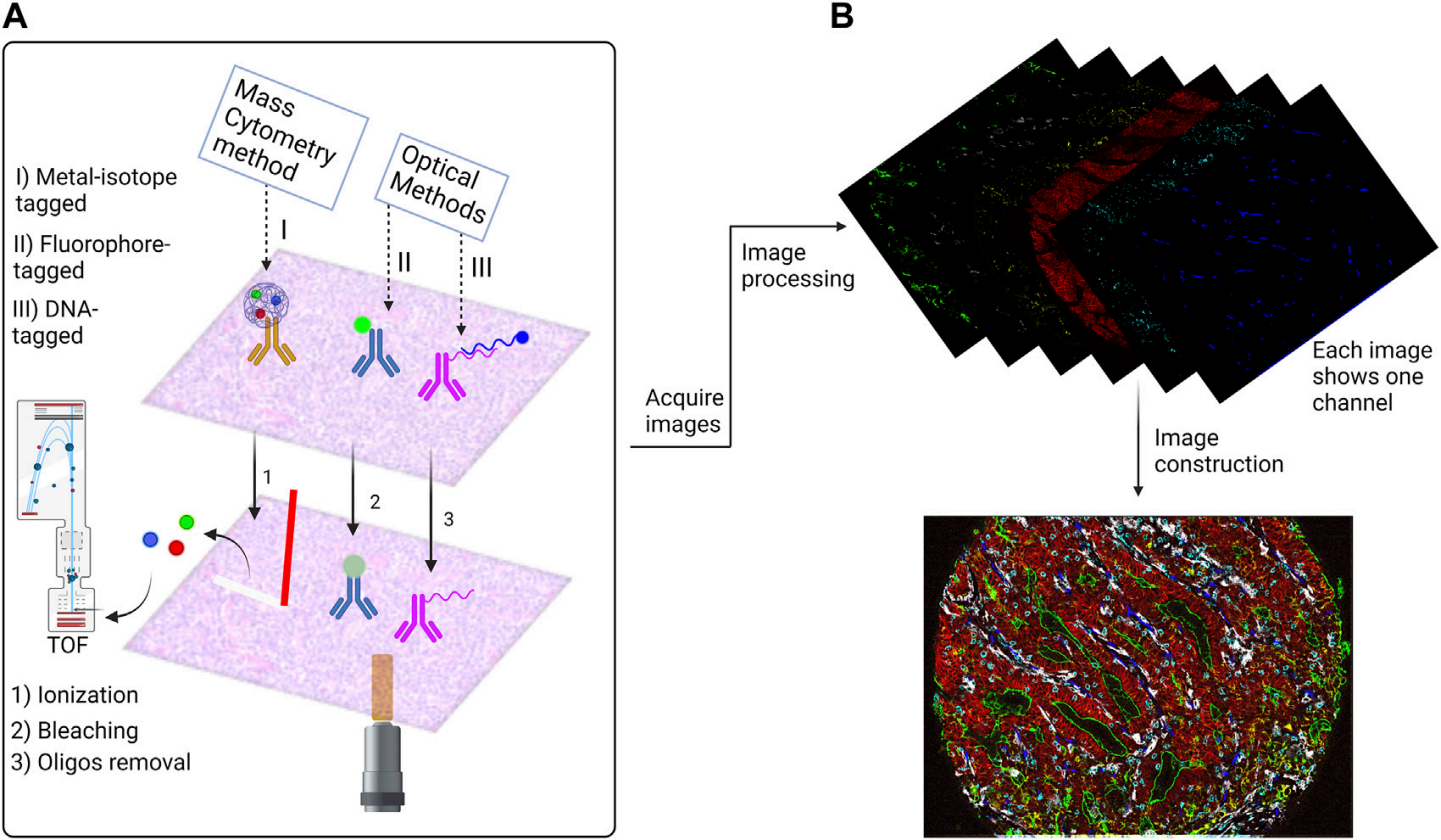
Overview of the antibody based Highly Multiplexed Tissue Imaging Techniques (HMTI). **(A)** HMTI techniques has two main categories: mass spectrometry methods which depend on conjugating the antibody with metal isotope and optical methods which depend on direct conjugation of a fluorophore to the antibody or conjugating it with oligo strand which is attached to a fluorophore. **(B)** An example of a highly multiplexed image. The outcomes of the optical methods are similar for the fluorophore and DNA-tagged methods; each cycle generates four images for four markers, one of them is nuclear stain which is used for the registration step. While the outcome of the metal isotope technique is a text file that contains the signal data, that is afterwards processed to generate a single image for each marker. The images then go through processing steps to denoising and stitching to construct a one virtual stacked image that contains all markers. The image shown is a colorectal cancer tissue imaged by Codex platform.

As shown in (Figure 1) the final outcomes of the two HMTI categories are similar as they are generating one single image for each marker. The difference is that the mass spectrometry-based imaging records the signal data in a text file that is processed to construct the marker images. The cyclic methods acquire separate images showing different markers for each channel of the microscope. Each cycle must contain the nuclear stain used for image registration to align all markers to the exact coordinates. Further processing steps are applied afterwards that include denoising, deconvolution, alignment and stitching. Eventually, the images are ready for advanced segmentation and analysis.

### Spatial transcriptomics

Single-cell RNA sequencing (scRNAseq) has lately made advances in the discovery of novel cell types and in our comprehension of how particular cell types influence health or react to alterations in surrounding microenvironments (Pham et al., 2020). scRNAseq is an extremely sensitive and thorough tool for classifying different types of cells (Svensson et al., 2017), as well as analyzing gene expression patterns (Kotliar et al., 2019) and developmental relationships (Haghverdi et al., 2016) along with transcriptional states to resolve individual cells (Pham et al., 2020). However, scaling it to millions of cells is prohibited by its high costs, and scRNAseq inherently lacks spatial resolution. However, gene expression in organs is spatially structured and varies in patterns (Biancalani et al., 2021). Therefore, spatial transcriptomics technologies have been developed that can combine data of gene expression and spatial location (Cutiongco et al., 2020) to identify differential expression (Svensson et al., 2018) or spatially dynamic genes (Dries et al., 2021). The ability to combine genome-wide transcriptional profiling of cells with data regarding tissue shape and spatial context opens up a world of possibilities for understanding cell biology in its original morphological and spatial environment (Pham et al., 2020).

### Deep learning

When it comes to artificial intelligence, DL is a subset of machine learning that refers to a deep artificial neural network, which is a particular structure of arranged artificial neurons in consecutive layers. The main two layers in a neural network are the first (called the input layer) and the last (called the output layer), while the layers between them are referred to as invisible or hidden layers. The first layer is the input (for example, slide or photo data). It has a set of parameters to produce the most accurate output. Each subsequent hidden layer gets input from the prior layer, applies its own parameters, and generates outputs. Finally, the last layer computes the overall model results.

**FIGURE 2:**
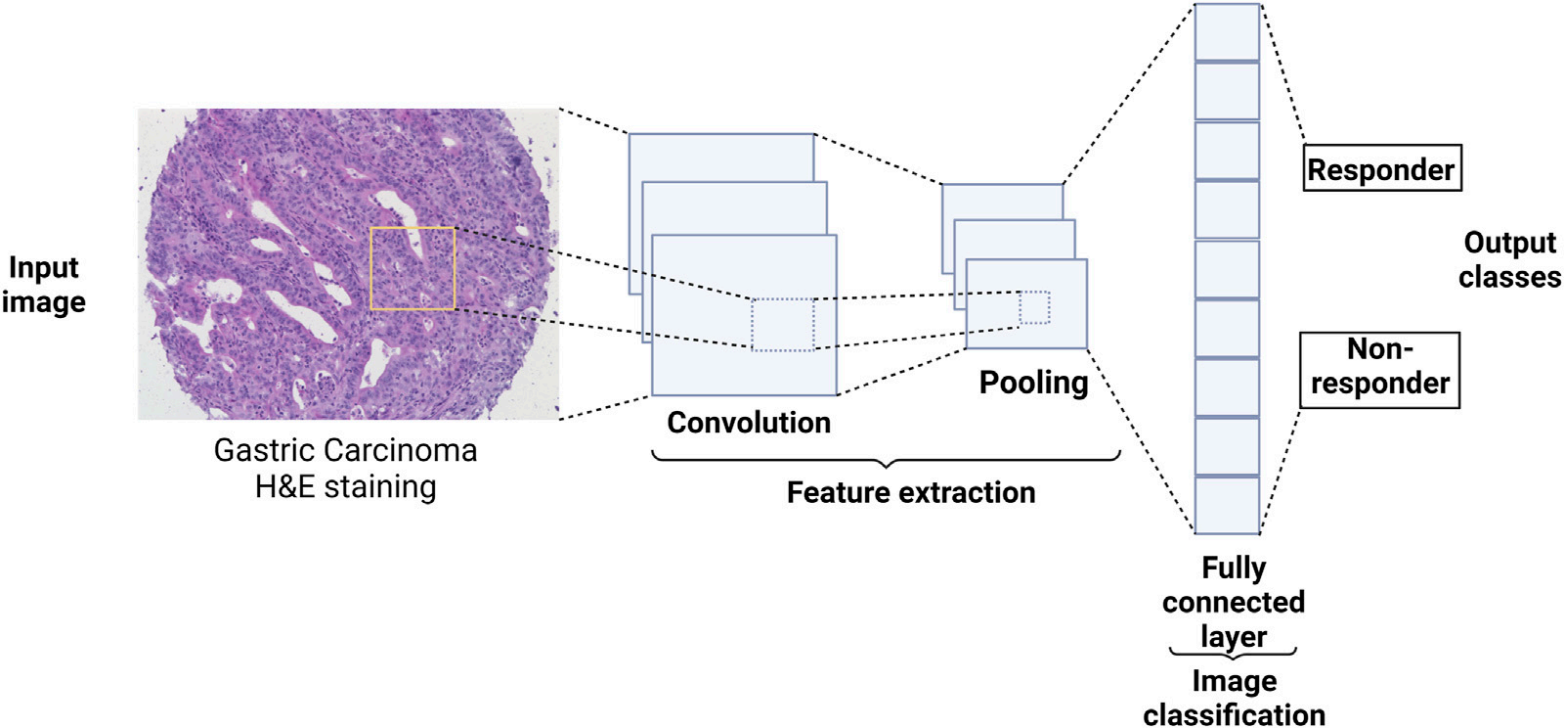
CNN architecture and how it could, conceptually, be utilized in medicine. A CNN starts with convolution and pooling layers which could be repeated several times to extract features from the image. The extracted features are then fed to a fully connected layer network where the class of the image is predicted. For examples of how CNN-based networks were used in medicine, see sections ‘Applications in conventional medical (“simple”) images’ and ‘Applications in highly multiplexed images'.

Several types of neural networks differ in their architecture. Our focus here is the convolutional neural network (CNN) because it is the core of the neural networks used in imaging data analysis discussed in this review. CNN undergoes two main tasks: feature extraction and class prediction. The former is a combination of convolution and pooling processes, while the latter is a fully connected network (Figure 2). The convolution process happens between a convolution kernel of size *p* × *p* and an area of the input image of the same size as the convolution kernel. The convolution process happens over the whole image by shifting the convolution kernel on the image. The kernel’s shift over the image is called ‘stride’. The stride value is defined by the number of pixels by which the kernel shifts over the image (e.g., stride = 1, means the kernel shifts by one pixel at a time). The convolution process happens in what is called the convolutional layer. A pooling layer follows the convolutional layer where image down-sampling happens to reduce the computational cost. The pooling layer has a kernel of size *n×n* which splits the image into smaller areas *k* of size *n* × *n* then takes the maximum pixel intensity value in each area *k* (maximum pooling) or the average of the pixel intensities in area *k* (average pooling). After the convolution and the pooling processes, the resultant image is flattened to represent the input of the second part of the CNN, which is a fully connected layer network. The fully connected layer network part ends with the output layer, which represents the number of possible classes “labels of the input image”. CNNs showed great performance in extracting the features of an image and hence very successful image classification (Goodfellow et al., 2016).

**FIGURE 3:**
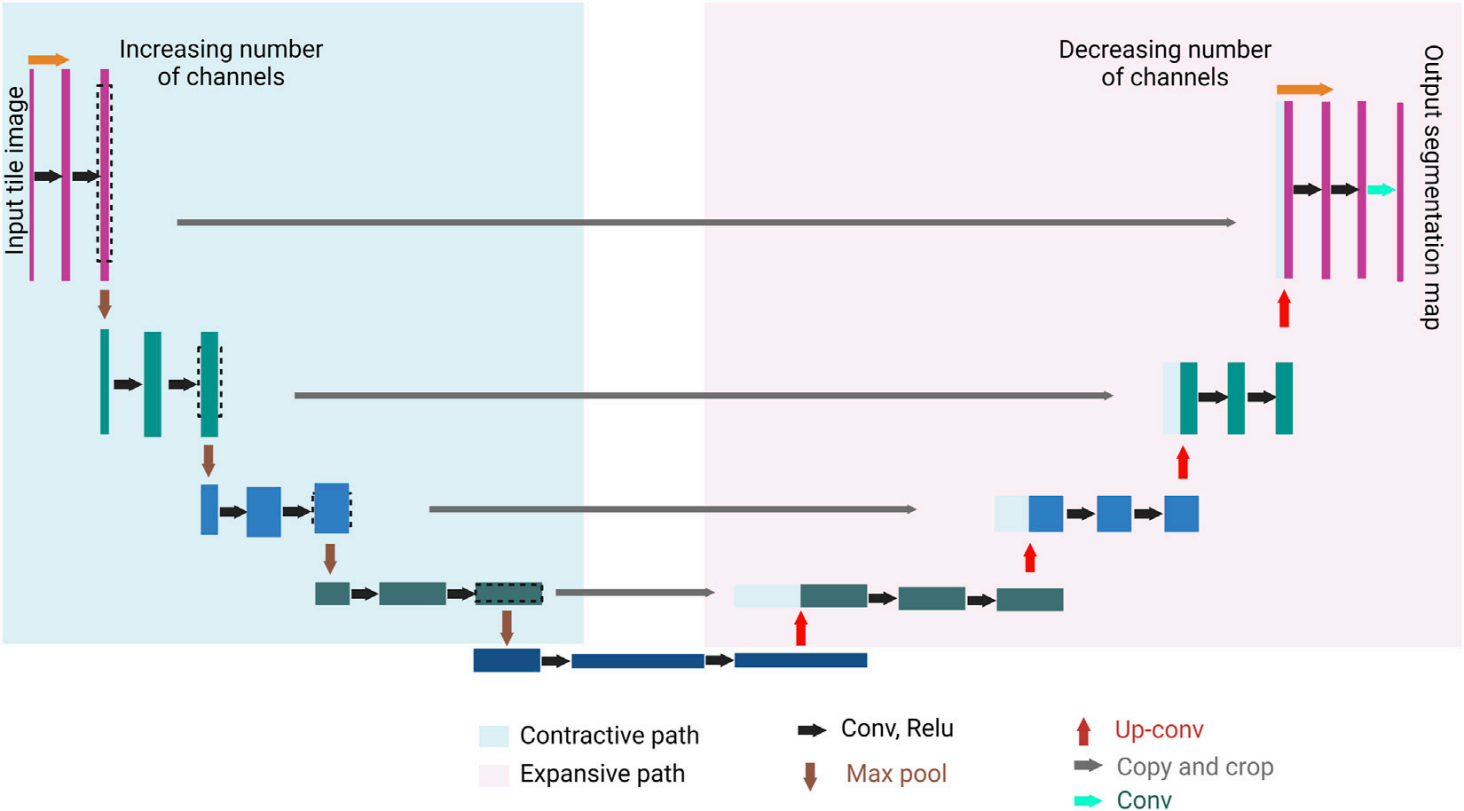
U-Net general architecture. U-Net consists of two paths, the contractive path (encoder) and the expansive path (decoder). A series of convolution processes (black arrows), and max pool processes (brown arrows) take place in the contractive path. The convolution process increases the depth of the image (number of feature channels increases), followed by a max pool process where the size of the image is halved. A series of up-convolution processes (red arrows), and concatenation processes (gray arrows) take place in the expansive path which ends with a convolution process (the green arrow). The up-convolution process halves the number of features channels, and the concatenation happens between the feature map from the expansive path and the correspondingly cropped feature map from the contracting path. The result of the U-Net is a semantically segmented image.

**FIGURE 4:**
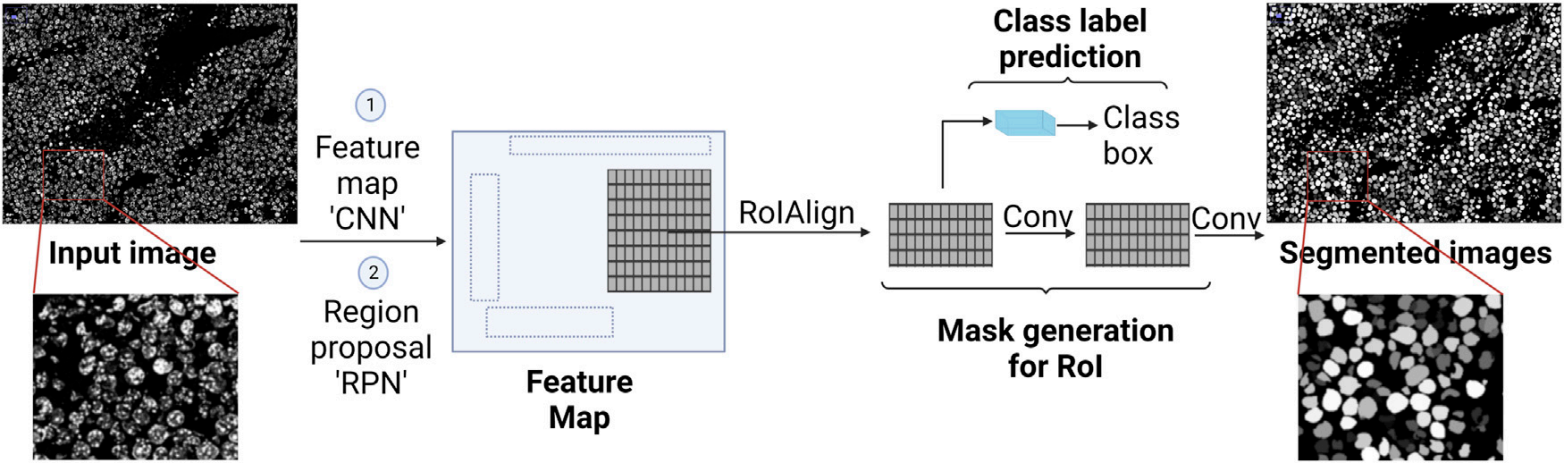
Illustration of Mask R-CNN and how it generates a segmented image (instance segmentation). It starts with convolutional layers to create a feature map, followed by region proposal network (RPN) for proposing regions of interest (RoI). Then the RoIAlign process takes place, where features are extracted from each RoI. Finally, masks are generated for each RoI using convolutional layers, and class prediction takes place using fully connected layers.

CNN is the cornerstone of a variety of neural network types such as U-Net and Mask Region-based (R)-CNN. Both networks are successful not only in extracting features from an image, but also in detecting objects in an image, hence, accurate image segmentation. U-Net has two main parts, forming a unique U-shape, the contractive path and the expansive path (Figure 3). Feature extraction happens in the former, while object detection occurs in the latter (Ronneberger et al., 2015). Mask R-CNN processes the images in three main stages (Figure 4): Feature map where feature extraction happens by a convolution-based network; region proposal, where object detection (region of interest, RoI) happens by a region proposal network (Ren et al., 2016); and mask head, where a mask is generated for each RoI by a convolution-based network (He et al., 2018). Both U-Net and Mask R-CNN perform object detection and assign each pixel in the image to a class label. However, there is a significant difference between them. U-Net treats objects of the same type as one entity (semantic segmentation), while Mask R-CNN treats objects of the same type as individual instances (instance segmentation).

### The need for deep learning in imaging data analysis

Histology slide images are quite informative: On a histology slide, millions of distinct cells may be viewed, and both the shape and the spatial arrangement of these cells reveal a wide spectrum of potentially crucial information. However, interpretation of such information can take a long time, requires highly skilled specialized personnel (doctors, pathologists, technicians), and is despite training of a specialist over several years still relatively error-prone. In synopsis with these elaborations, it is essential to create diagnostic methods that are efficient yet affordable, which could be fulfilled using DL (Yu et al., 2021).

The uniqueness of DL is its capacity to learn sophisticated representations to enhance pattern recognition from unprocessed data and has shown a capacity to revolutionize diagnosis in medicine. There are a multitude of instances showcasing how DL contributed to the growth and advancement of pathological research (Komura and Ishikawa, 2018), such as analysis of malignancies of the lung (Hua et al., 2015), lymph nodes (Ehteshami Bejnordi et al., 2017), skin (Esteva et al., 2017), and colorectum (Yu et al., 2021). There are also many instances demonstrating how DL algorithms can exploit input data from biological images for the determination of patient therapy, including radiographic imaging, magnetic resonance imaging, and positron emission tomography (Shen et al., 2017). In single-cell optical image research, DL was effectively used for image identification, further classification and segmentation (Min et al., 2017), cell imaging system design and control, cross-modal and super-resolution image reconstruction, cell tracking, and quantification (Sun et al., 2020). Technological breakthroughs in DL research applied on millions of images have proven equal precision to assessments by board-certified clinical professionals (De Fauw et al., 2018; Hekler et al., 2019; Qian et al., 2021). Therefore, computer-assisted diagnostics have started to be advantageous for academics and physicians (Shen et al., 2017).

### The need for deep learning in spatial “omics” technologies data analysis

**FIGURE 5:**
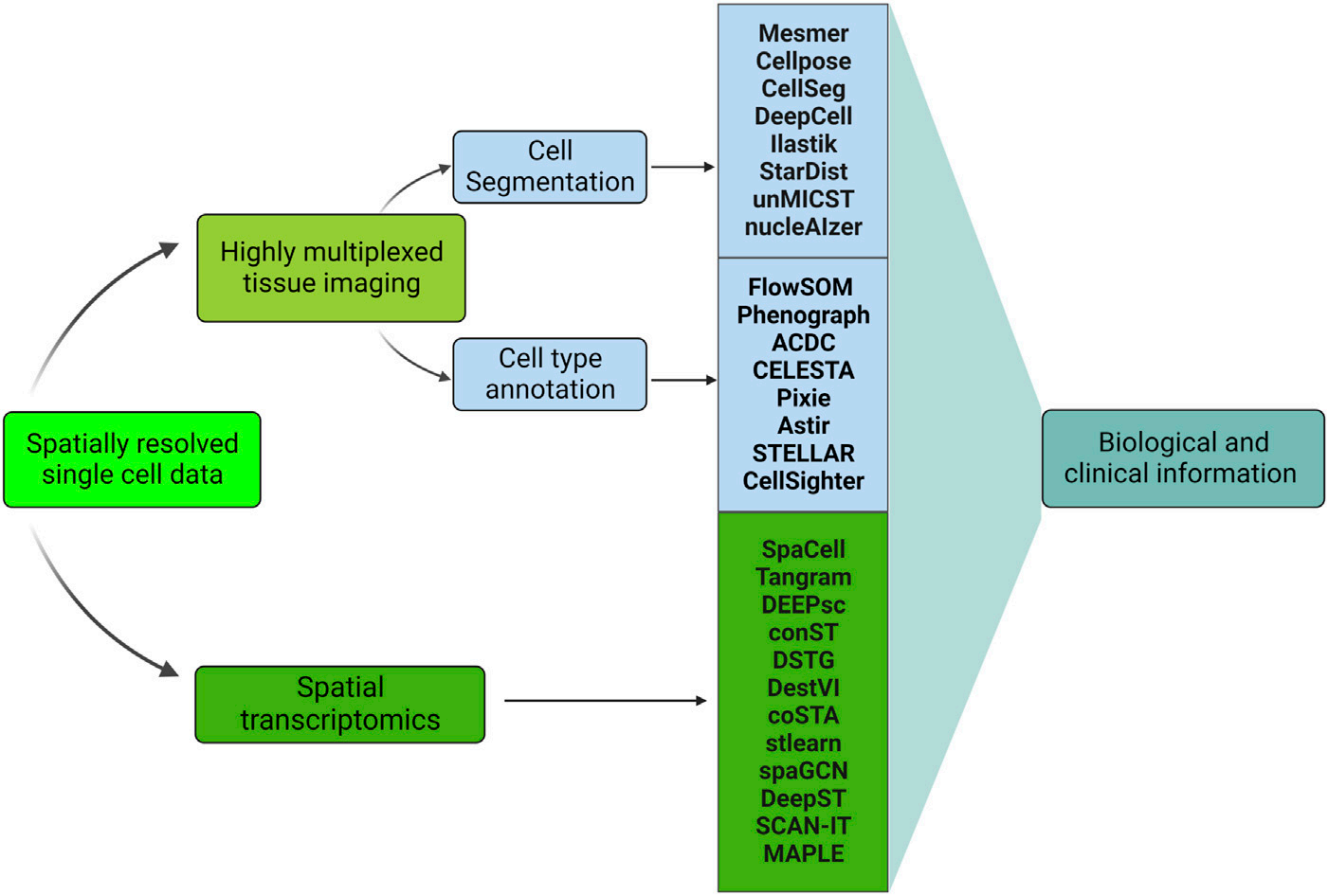
Machine learning-based pipelines used in analyzing spatially resolved single-cell data. All the methods are DL-based except ilastik, Phenograph, ACDC, and CELESTA. Highly multiplexed tissue imaging and spatial transcriptomics represent the two main categories of spatially resolved single-cell data. DL is involved in the highly multiplexed images’ preprocessing (cell segmentation and cell type annotation), see sections ‘Deep Learning in cell segmentation’ and ‘Deep Learning in cell type annotation’. In addition, DL is utilized for spatial transcriptomics data analysis, see section ‘Deep Learning in spatial transcriptomics’. Both Highly multiplexed tissue imaging and spatial transcriptomics analysis leads to obtaining biological and clinical information, see section ‘Applications in Image Analysis’.

Cutting-edge machine learning algorithms, applied to new types of datasets generated by genomics techniques, integrate image pixel data with molecular analysis to define tissue architecture in images surpassing the limitations of conventional pathological labeling (Hekler et al., 2019). Large scale implementations of DL development have been achieved in the studies of gene expression and protein structure prediction (Lyons et al., 2014). We will briefly cover the most widely used DL-based pipelines in spatial transcriptomics analysis in ‘Deep Learning in Spatial Transcriptomics’. Figure 5 summarizes how DL is involved in analyzing HMTI and spatial transcriptomics data.

In the following sections, we focus on cutting-edge DL methods developed for HMTI data analysis, including methods for image segmentation, cell type annotation, tissue analysis, and DL methods for spatial transcriptomics.

## Applications in image preprocessing

Image preprocessing includes two main steps: image segmentation and cell type annotation. Here we discuss how DL enhances these two steps.

### Deep learning in cell segmentation

Image segmentation is one of the key tasks in computer vision with many applications in several fields, specifically digital pathology. It could be seen as a classification problem of the pixels in an image (Minaee et al., 2020). There are two main types of image segmentation: Semantic segmentation, where the pixels are classified with labels (e.g., Pyramid Scene Parsing Network ‘PSPNet’ (Zhao et al., 2017) and UNet-based networks (Ronneberger et al., 2015)) and instance segmentation where, the individual objects (e.g., nuclei) are labeled (e.g., Mask R-CNN (He et al., 2018)) (Minaee et al., 2020). For digital pathology and HMTI, accurate segmentation of cells is a crucial step because it determines the accuracy of the downstream analysis. DL-based tools are useful for image segmentation due to their excellent performance (Luo et al., 2021). Here, we discuss several DL-based segmentation pipelines (summarized with pros and cons in Table 1), how each pipeline tackles a specific problem in image segmentation, and whether it is trained on single-stained, conventional medical (“simple”) images or highly multiplexed images. In addition, we show that even if the pipeline was trained on “simple” images, it could be used for highly multiplexed images, paving the way for the downstream analysis to obtain mechanistic insights from the collected data.

**TABLE 1:** The core learning approach/model of cell segmentation and cell type annotation pipelines and the corresponding advantages and disadvantages.

Method	Machine learning approach	Advantages	Disadvantages	References
Segmentation pipelines				
Mesmer	feature pyramid network	Generalization	Inaccurate image segmentation in cases of low signal-to-noise ratio, heterogeneous staining, and focus issues	Greenwald et al. (2022)
		No manual parameter tuning		
		Fast		
		Retraining is not required		
Cellpose	U-Net with residual blocks.	Generalization	Low convexity cells are not well segmented	Stringer et al. (2021)
		No parameter adjustments		
		Retraining is not required		
		3D cell segmentation using the 2D model and without new 3D-labeled data		
		Cell tracking extension		
Cellseg	Mask R-CNN	Generalization	Parameter tuning is required, in a limited level though	Lee et al. (2022)
		Retraining is not required		
		Pixel quantification		
		Designed as a library, enabling customizations		
DeepCell	Deep CNN	Robust identification of a cell’s cytoplasm with single cell resolution obtained from phase microscopy	Requires prior training on new cell types	Van Valen et al. (2016)
Ilastik	Machine learning based non-linear classifier.	Interactive tool	ilastik does not include an option to train deep convolutional neural networks (CNNs)	Berg et al. (2019)
		Does not need large training data. ilastik can handle data in up to five dimensions (3D, time and channels)		
		ilastik ImageJ plugin availability		
StarDist	U-Net	Pretrained	In case of wrong segmentation, sometimes StarDist omits cells	Schmidt et al. (2018)
		Could be combined with TrackMate (Ershov et al., 2021) for cell tracking		
		Accurate for images with crowded cells		
		Good for segmenting images of irregular morphologies		
UnMICST	UNet, Mask R-CNN, PSPNet	Segmenting images with artifacts (blurring, out of focus)	Model does not learn subtle shape and texture differences between cell types	Yapp et al. (2022)
Cell type annotation Pipelines				
FlowSOM	Self-organizing map	Useable for visualization	Not interpretable	Van Gassen et al. (2015)
PhenoGraph	Graph-based clustering, community detection	Scalability	Manual parameter selection	Levine et al. (2015)
ACDC	Knowledge transfer-based clustering	Incorporates expert knowledge	Relies on user-defined cell types	Lee et al. (2017)
CELESTA	Knowledge-driven score-based label assignment	Incorporates expert knowledge, uses spatial information	Relies on user-defined cell types, parameter fine tuning for rare cell types	Zhang et al. (2022)
Pixie	Self-organizing map, hierarchical clustering	Independent of segmentation	More elements must be clustered	Liu et al. (2022)
Astir	Deep recognition neural network	Incorporates expert knowledge, Interpretable, identifies unspecified cell types	Does not consider spatial information	Geuenich et al. (2021)
STELLAR	GCN	Includes spatial information	Requires References data set	Brbić et al. (2022)
CellSighter	CNN	Probabilistic output	Supervised learning requires all cell types to be present in training data	Amitay et al. (2022)

Several models were assessed with respect to their generalization performance, i.e., here, ‘the segmentation performance on test data not available for model training’. One of them being Mesmer (Greenwald et al., 2022), a DL-based pipeline that was trained on a unique dataset called TissueNet. The TissueNet dataset was obtained from diverse imaging platforms (CODEX, Cyclic Immunofluorescence, IMC, MIBI, Vectra, and MxIF), including various disease states and tissue types. Furthermore, it is a comprehensive segmentation dataset which consists of paired nuclear and whole-cell annotations, which, when combined, sum up to more than one million paired annotations (Greenwald et al., 2022). Moreover, TissueNet has 16 times more whole-cell annotations and twice as many nuclear annotations than other datasets (Greenwald et al., 2022). Mesmer was able to predict diagnostically relevant features including the subcellular localization of proteins in cells. Hence the quantity of nuclear translocation of transcription factors, as well as the degree of membrane staining of HER2, could be measured which in turn could be used for breast cancer assessment (Greenwald et al., 2022). Mesmer was used in several studies to segment highly multiplexed images, leading, after downstream analysis, to several discoveries. Instances are the demonstration of how uninvolved lymph nodes (i.e., no regional lymph node metastasis) can provide response hallmarks in regard to anti-tumor immune therapy in human head and neck carcinoma (Rahim et al., 2023) and showing that there are distinct architectural tumor microenvironment (TME) states in the transition from ductal carcinoma *in situ* (DCIS) to invasive breast cancer with certain features being ascribed as protective against recurrence (Risom et al., 2022).

Following the same concept of training models on diverse data types, Cellpose, another segmentation pipeline, was trained on two categories of data: images of cells and images of nuclei (Stringer et al., 2021). In addition to that, the training dataset included images from other microscopes and repeated objects, such as jellyfish, rocks, and fruits. The inclusion of diverse images was designed to make Cellpose generalize more robustly. Furthermore, Cellpose has spare capacity for additional training data, i.e., contributing more training data will not lead to saturating the learning capacity of Cellpose. The authors also introduced Cellpose3D, which can perform 3D segmentation after being trained on 2D data. Cellpose3D could be directly trained on 3D ground truth data and additionally be extended to perform other tasks as cell tracking (Stringer et al., 2021). Moreover, a SpatialVizScore that quantifies the immune cell infiltration in lung tumor samples, was developed based on Cellpose segmentation (Allam et al., 2022).

Another segmentation pipeline that features generalization is Cellseg, which is a Mask R-CNN based software capable of cell segmentation and pixel quantification. CellSeg is among the most accurate pipelines which were tested on the 2018 Kaggle Data Challenge. CellSeg does not need training as it is a pre-trained model. Initially, CellSeg was trained on brightfield and fluorescence microscopy images provided from the 2018 Kaggle Data Science Bowl. Without being trained on any highly multiplexed images, CellSeg was able to segment 140 colorectal cancer images from a CODEX study (Lee et al., 2022). Furthermore, CellSeg identifies tumor cells with higher sensitivity because it does not over-segment the large tumor cell nuclei as a commonly used watershed algorithm does (Lee et al., 2022). CellSeg was part of several discoveries, such as showing that the spatial cellular ecosystem which controls muscle regeneration changes with aging (Wang et al., 2022), identifying the functional and cellular properties of tertiary lymphoid structures which provide therapeutic clues for cancer and autoimmunity (Nayar et al., 2022), and identifying entity-specific spatial and quantitative aberrations of the T-cell microenvironment in nodal B-cell lymphoma (Roider et al., 2022).

Another segmentation pipeline featuring generalization is DeepCell which enables nucleus and cytoplasm segmentation of individual phase-contrast images of mammalian cells without requiring fluorescent signals of cytoplasmic markers (Van Valen et al., 2016). The significance of DeepCell is that it enables robust identification of a cell’s cytoplasm with single-cell resolution obtained from phase-contrast which used to be a difficult task. Although DeepCell has a generalizability feature, it requires prior training on new cell types (Van Valen et al., 2016). Applying DeepCell for segmentation of HMTI data enabled findings such as associating functional proteins of cell-cell interaction with recurrence and overall survival predictions of triple-negative breast cancer (Patwa et al., 2021), and investigating the intra-patient tumor organization heterogeneity in triple-negative breast cancer (Keren et al., 2018).

To enhance the segmentation performance, Ilastik was developed as an interactive tool that allows the user to click on wrongly classified or uncertain positions, introduce labels, annotations or sparse training examples, hence retrain the classifier on a bigger training set that includes both the old and the new user labels. Ilastik provides a fast-learning process as a refinement system within a timeframe smaller than the time needed for dense ground-truth annotation (Berg et al., 2019). Furthermore, Ilastik includes multiple workflows such as pixel classification and tracking overflow (Berg et al., 2019). Ilastik was utilized in analyzing HMTI data leading to several biological discoveries. For instance, unraveling cancer-associated fibroblasts (CAF) heterogeneity and investigating TME remodeling in an immunocompetent mouse orthotopic lung cancer model (van Maldegem et al., 2021).

One of the issues in segmentation is the occurrence of objects with different morphologies. StarDist approaches this problem by training a convolutional neural network (U-Net) to predict a star-convex polygon (instead of a bounding box) for each pixel (only for non-background pixels of an object) in the image for the cell instance at a specific position. The advantage of this polygon-based method is that it can cope with several shapes and efficiently segment images with very crowded nuclei. It is worth mentioning that the mistakes made by StarDist are handled either by omitting a particular cell or by predicting a plausible cell shape (Schmidt et al., 2018). In contrast, mistakes made by other segmentation methods potentially lead to implausible outcomes (Schmidt et al., 2018). An example of StarDist application to HMTI data is segmenting high-grade serous ovarian cancer (HGSOC) images leading to immune recognition and evasion investigations (Vázquez-García et al., 2022).

Many segmentation pipelines focus on adjusting the model architecture to optimize performance. However, universal Models for Identifying Cells and Segmenting Tissue (UnMICST) was developed to enhance the performance by manipulating the input data. UnMICST is a family of neural networks, each being trained separately (Yapp et al., 2022). UnMICST comprises UNet, Mask R-CNN, and the Pyramid Scene Parsing Network (PSPNet). The authors showed that the segmentation accuracy is improved by manipulating the input rather than the network’s architecture (Yapp et al., 2022). To this end, two ways were shown to improve segmentation accuracy. First, adding nuclear envelope staining (NES) images to images of nuclear chromatin obtained by DNA-intercalating dyes. Second, real augmentation which is the process of intentionally oversaturating (by means of long exposure time) and defocusing the images to mimic the artifacts happening in real tissue imaging so that the trained models are more robust. The results showed that real augmentation outperforms augmentation by conventional Gaussian blurring (Yapp et al., 2022). Furthermore, training the models on data including real augmented data and NES data was shown to have a cumulative effect. Interestingly, the cumulative effect could be observed across different tissue types (Yapp et al., 2022). To test the robustness of the trained UnMICST on segmenting highly multiplexed images, 64-plex CyCIF images of non-neoplastic small intestine tissue were fed into the UnMICST. Segmentation masks were accurately located and almost no under- or over-segmentation was detected (Yapp et al., 2022). In addition, low abundance (3%) of CD45 and E-cadherin double-positive cells was detected, reflecting accurate segmentation due to mutual exclusion. Investigation of CD45^+^ E-cadherin + cells revealed that some were CD3^+^ T-cell which were in close proximity to or between epithelial cells of the intestinal villi. This observation aligns with the intestinal epithelium’s known role in immune homeostasis. In such cases, humans can distinguish between the epithelial and immune cells based on subtle shape and texture differences, as well as multi-dimensional intensity features which are not featured in the model training. Therefore, developing a model that is aware of physiology could allow the recognition of biologically relevant features (Yapp et al., 2022). Other studies segmented their data using UnMICST to investigate immune evasion and immunoediting in primary melanoma (Nirmal et al., 2022) and the purinergic signaling topology in glioma (Coy et al., 2022).

Finally, nucleAIzer was developed to segment cells based on the image style transfer concept combining a Mask R-CNN-based instance segmentation network and a U-Net-based semantic segmentation network to provide a robust segmentation of a wide variety of cell types from different staining methods and diverse image modalities (Hollandi et al., 2020). To our knowledge, nucleAIzer was not yet used to segment HTMI data.

### Deep learning in cell type annotation

A central aspect of single-cell analysis in spatial and non-spatial contexts is the cell type annotation of measured cells. This task is challenging because single-cell data suffers from high dimensionality, noisiness, and technical artifacts. With the rise of machine learning and DL in the past years, various algorithmic approaches have been proposed to overcome this challenge. Current research mainly uses manual annotation of cells using domain knowledge, which is considered the gold standard for this task.

The proposed methods for cell type annotation (summarized with pros and cons in Table 1) can be divided into two groups based on the machine learning paradigm they are based on: unsupervised, as in clustering, and supervised, as in classification. While supervised annotation tools require ground-truth labels for training, unsupervised methods are purely data-driven, although they might consider prior knowledge.

Established procedures for cell-type annotation have been applied to studies using spatially resolved data and are still used. As suspension-based single-cell data is the predecessor of spatially resolved single-cell data, many computational methods designed for suspension-based single-cell data have later been applied to spatially resolved single-cell data. Only recently, specialized methods accounting for spatial information have been developed. Thus, we will first give an overview of methods used for non-spatial data, followed by more recent approaches.

Prior to the development of distinguished methods for single-cell data, classical machine learning approaches for clustering have been used, including K-means clustering and graph-based Louvain clustering. Generally speaking, community detection on graphs has become the method of choice for clustering single-cell data (Luecken and Theis, 2019).

FlowSOM was one of the earliest tools designed explicitly for cell-type annotation in cytometry data (Van Gassen et al., 2015). It is based on a self-organizing map (SOM) and is thus a DL-based clustering method. Levine et al. proposed a graph-based approach, PhenoGraph, which extends established community detection methods like Louvain clustering to detect rare phenotypes more accurately (Levine et al., 2015). This is achieved by refining the underlying graph to account for shared neighbors of nodes.

ACDC. proposed by Lee et al. (Lee et al., 2017), allows the user to incorporate prior knowledge about marker expression to define a matching score used for clustering. Following these and other methods engineered to be suitable for phenotyping single cells, Abdelaal et al. suggested linear discriminant analysis (LDA) for classification, thus using a supervised learning approach (Abdelaal et al., 2019). The authors have shown that LDA outperforms more complex methods in this task on chosen datasets.

CELESTA. Although research on spatially-aware cell type annotation tools has just started, a few methods are already available. Zhan et al. presented CELESTA, a method that leverages a cell-type signature matrix to assign cell-type probabilities to each cell (Zhang et al., 2022). In the first step, so-called “anchor cells” are defined based on a marker-scoring function. Subsequently, the remaining cells are assigned to cell types based on a combined marker- and spatial-scoring function in an iterative fashion. The algorithm converges after a specified threshold of identified cells is reached.

Pixie. developed by Liu et al., annotates cell types in highly-multiplexed *in-situ* imaging by clustering each pixel of the provided image (Liu et al., 2022). The method combines self-organizing maps and consensus hierarchical clustering to assign classes to each pixel while allowing for manual interventions. These pixel classes are then used as features of each segmented cell for assigning each segmented cell to a cell type.

Astir. proposed by Geuenich et al. for automated probabilistic cell-type assignment (Geuenich et al., 2021). While the authors apply the method for both suspension expression and highly multiplexed imaging data, it is of note that Astir does not account for spatial information provided by the imaging data.

STELLAR. a pipeline based on a graph convolutional neural network (GCN), that was proposed by Brbić et al. (Brbić et al., 2022). The method requires annotated data to be trained, putting it in the supervised learning category. The method uses a k nearest neighbor graph with marker expression as node features. The authors demonstrate that the generated embeddings can potentially be interpreted as spatial modules.

CellSighter is another neural network-based approach proposed by Amitay et al. (Amitay et al., 2022). CellSighter is based on multiple convolutional neural networks (CNNs) which operate on the provided imaging data. The model accounts for uncertainty by assigning each cell a probability for all available classes. Each CNN input is a 3-dimensional tensor containing images of K proteins centered in the cell. As additional input, two images are provided. The first image encodes the segmentation of the cell to be classified, while the second image contains similar information but also includes neighboring cells.

## Applications in image analysis

**FIGURE 6:**
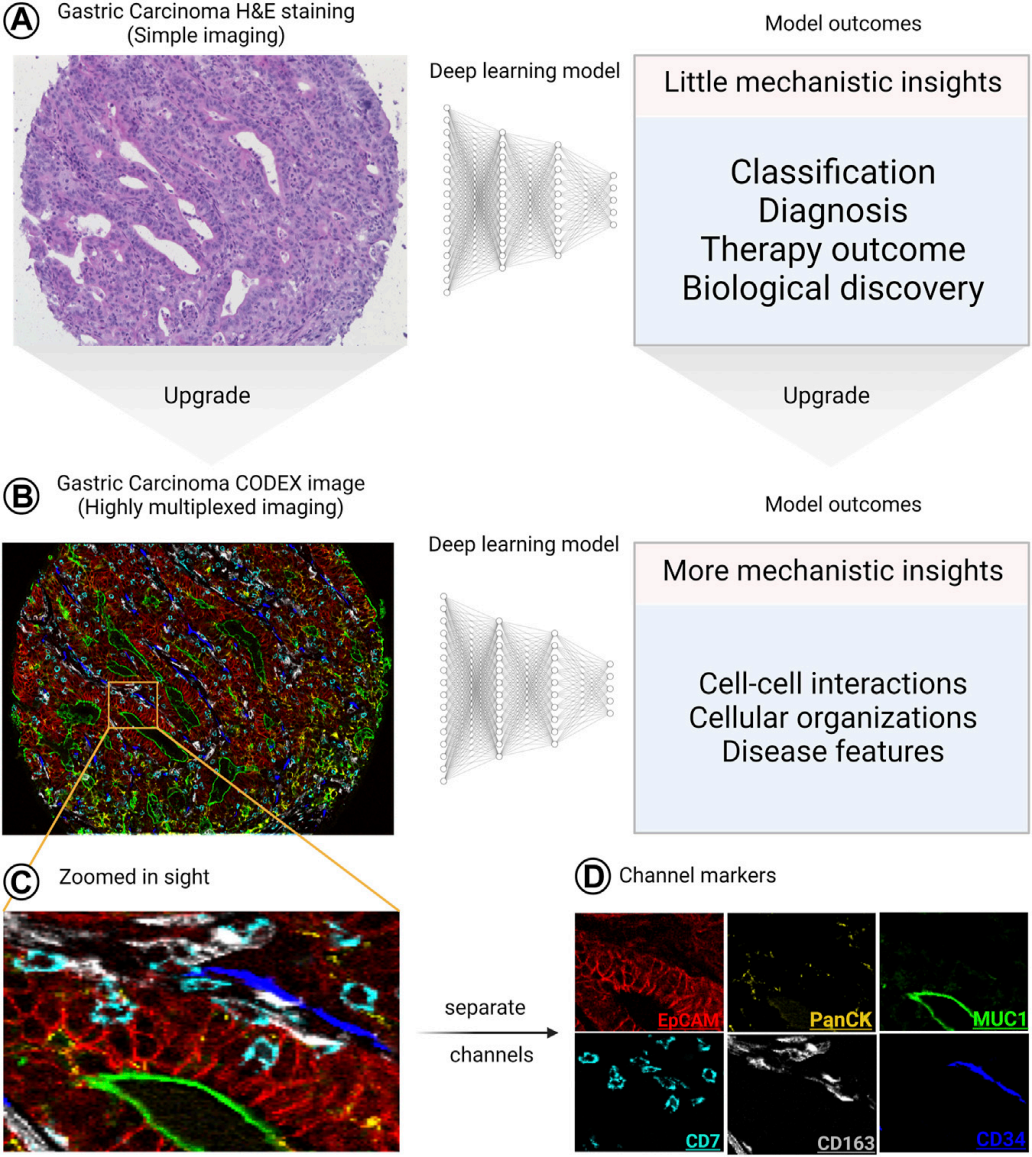
The significant upgrade of the outcomes of combining HMTI with DL. **(A)**, when “simple” imaging (like hematoxylin and eosin [H&E]-stained slides) is combined with DL, the outcomes provide little mechanistic insights. **(B)** On the other hand, when a highly multiplexed image is analyzed using DL, the provided outcomes do not only cover what “simple” images cover but also provide more detailed mechanistic insights. **(C–D)** Zoomed in sight for an area of the image showing *6* channels as **(C)** overlay and **(D)** separate images, where each channel is defined by a specific marker. The H&E and CODEX images are reproduced from (Schürch et al., 2020) under a CC-BY license.

As shown in “Deep Learning in Segmentation”, upgrading the segmentation pipelines to be used on highly multiplexed images enabled biological discoveries with mechanistic insights that could have never been obtained if “simple” imaging was used. In this section of the review, we elaborate more on this concept by giving examples of what could be obtained from combining DL with “simple” images and combining DL with highly multiplexed images. We conclude that the biological information obtained from the combination of DL with “simple” images is limited to disease diagnosis, classification and therapy outcome predictions with little mechanistic insights even with the aid of explainable AI tools. However, if “simple” images are replaced with HMTI, the same questions are more precisely addressed and accompanied by a multitude of additional insights, as summarized in Figure 6.

### Applications in conventional medical (“simple”) images

Classification. DL was effectively used for medical image classification and segmentation (Hashemzadeh et al., 2021), showing a considerable potential in pathological image analysis, such as tumor and metastasis localization (Wang S. et al., 2019). The successful applications of DL were expanded to cover brain tumor identification (Hossain et al., 2019), breast cancer research (Ragab et al., 2019), segmenting gastrointestinal malignancy (Yoon and Kim, 2020), classification of lung tumors (Alakwaa et al., 2017), prostate cancer detection (Yoo et al., 2019), and many more. For instance, ResNet-101 (He et al., 2015) was trained on multi-phase computed tomography images to classify the renal tumors into their major subtypes (Uhm et al., 2021); clear cell renal cell carcinoma (RCC), papillary RCC, and chromophobe RCC, angiomyolipoma and oncocytoma, where the first three are malignant and the last two are benign (Moch et al., 2016; About Kidney Cancer, 2023). In another study, microfluidic technology was combined with DL algorithms to classify between five distinct lung cancer cell lines and a healthy cell line (Hashemzadeh et al., 2021). The authors have trained five CNN models, ResNet18, SqueezeNet (Iandola et al., 2016), AlexNet (Krizhevsky et al., 2017), GoogLeNet (Szegedy et al., 2015), and Inceptionv3 (Szegedy et al., 2016), which were pre-trained on ImageNet (Deng et al., 2009). ResNet18 outperformed the other networks (Hashemzadeh et al., 2021). In another work, DL extracted decisive features from ultrasound (US) images to predict the risk of breast malignancy (Qian et al., 2021). The images were bimodal and multimodal US images, including ultrasound elastography images, ultrasound color Doppler and US B-mode (Qian et al., 2021). The DL model was an integration of ResNet-18 with SENet (Hu et al., 2019) backbone (Qian et al., 2021). The model was pre-trained on ImageNet dataset via transfer learning (Qian et al., 2021). In addition, the gradient-weighted class activation mapping (Grad-CAM) (Selvaraju et al., 2020) provided the explainability to the prediction, which assists the clinicians in comprehending the classifications made by the model (Qian et al., 2021).

Yu, G. et al. built a semi-supervised learning (SSL) model that classifies colorectal cancer samples into cancerous and noncancerous based on whole slide images (Yu et al., 2021). SSL with a small amount of labeled data outperformed the supervised Learning model (SL) with larger labeled data (Yu et al., 2021). This was proven at both patient-level and patch-level colorectal cancer (Yu et al., 2021). The model was trained on lung and lymph nodes cancer datasets, which showed the superiority of SSL over the SL (Yu et al., 2021). At the patch-level, the SL model is built on Inception V3 (Yu et al., 2021). While the baseline for SSL model employs two Inception V3, the first one is called teacher and the other one is called student. This method is named the mean teacher method (Tarvainen and Valpola, 2018). At the patient-level, the inception V3 network was trained on the pathological images after pre-training on ImageNet dataset (Yu et al., 2021).

The success of DL continues with the histological images. A DL-based model analyzed the cellular morphology of H&E images to create a classifier that predicts the breast cancer grade, estrogen receptor status, histologic subtype (lobular or ductal tumor), histologic subtype (lobular or ductal tumor), gene expression score and the risk of recurrence score (Couture et al., 2018). The authors applied CNN and VGG16 architectures (configuration D) (Simonyan and Zisserman, 2015) for image pre-processing and features identification. The VGG16 was pre-trained on the ImageNet dataset. A probabilistic model was exploited to determine the class of each group. A linear support vector machine (SVM) calibrated with isotonic regression was employed to predict these probabilities (Couture et al., 2018).

Therapy outcomes. DL successfully predicted therapy outcomes for several tumors using data obtained from different modalities. For instance, a multi-task DL strategy which exploits the dynamic information in longitudinal images, was used to predict tumor response to treatment (Jin et al., 2021). The multi-task learning framework is based on a 3D RP-Net architecture (Wang L. et al., 2019) consisting of two parts: one of them being feature extraction and tumor segmentation, which is a convolutional encoding/decoding subnetwork, and a response prediction part which is a multi-stream Siamese subnetwork. The framework was trained on magnetic resonance imaging scans of rectal cancer patients to predict the pathologic complete response (pCR) after neoadjuvant chemoradiotherapy. Network visualization highlighted that the extramural vascular invasion and the depth of tumor invasion are correlated with the poor response of the patients (Jin et al., 2021); however, that does not guide well to mechanistic insights.

Other work demonstrated anti-VEGF therapy outcomes on metastatic colorectal cancer patients by using a DL-based framework only trained on computed tomography (CT) scan images obtained from the VELOUR trial (ClinicalTrials.gov identifier NCT00561470), and combined with the standard tumor size-based evaluation methodology. Interestingly, when the DL-based method was used without a combination of the standard tumor-based method, the DL-based framework still performed better than the standard tumor-based method (Lu et al., 2021). The DL-based framework consists of two neural networks: a convolutional neural network CNN (pre-trained GoogleNet on ImageNet), which was trained on the CT scans to extract the important features from lesions of different organs, and a recurrent neural network RNN which learned the changes happening in these lesions across multiple time points. Furthermore, Grad-CAM was used to find out the regions of the input CT scans that contributed the most to the framework predictions. However, further investigations are required to understand the underlying biology of these significant regions (Lu et al., 2021).

Other studies investigated the early postoperative recurrence in intrahepatic cholangiocarcinoma patients. CT scans were used from a pilot study of intrahepatic cholangiocarcinoma patients to train a residual neural network (ResNet50) (He et al., 2015)). The pilot study contained patches collected from patients with and without early recurrence. The DL approach preoperatively predicted the recurrence risk after surgery (Wakiya et al., 2022).

Previously, DL-based frameworks were able to predict the ERBB2 (HER2) status based on learning tissue architecture from digitized H&E-stained specimens (Shamai et al., 2019). An extension of this was training a DL model on H&E-stained formalin-fixed primary breast tumor tissue images (weak supervision by ERBB2 amplification), given that the ERBB2 gene amplification was detected using chromogenic *in situ* hybridization. The predicted ERBB2 amplification was called the H&E-ERBB2 score and was correlated with distant disease-free survival. Besides a fully connected block, the framework consists of a stack of layers from se-resnetxt50_32 × 4d, ‘a squeeze-and-excitation convolutional neural network architecture’ (Hu et al., 2019). The neural network’s weights were the ones trained on ImageNet. Interestingly, there was a significant variability of the H&E-ERBB2 score across and within the samples indicating a heterogeneous distribution of the ERBB2-associated patterns in the tissues. Therefore, Grad-CAM activation maps were used to find the regions of the tissue which are the most predictive of the ERBB2 gene amplification. The Grad- CAM activation maps showed that the regions of *in situ* carcinoma components and the tumor epithelium are the most predictive. In addition, this study showed that there is an association between breast cancer survival and some features, such as the distance between the tumor regions, the stroma-tumor interface, and the size of the tumor nests. However, highly multiplexed techniques are required for further investigations (Bychkov et al., 2021).

Finally, a DL model was trained on quantitative ultrasound multiparametric images of breast cancer to predict the neoadjuvant chemotherapy response. This study tested the performance of two different neural network architectures (residual network and residual attention network) using two different experiments. In the first experiment, the feature map was extracted only from the tumor core, while in the second experiment, the feature map was extracted from both the tumor core and its margin. It turned out that the best performance was obtained from the RNN architecture with the feature map including both the tumor core and its margin (Taleghamar et al., 2022).

Biological discovery. Many instances of machine learning have been applied to “simple” imaging techniques for biological discovery. Saltz et al. detected tumor necrosis and tumor-infiltrating lymphocytes on H&E stainings. They achieved this by using a CNN-based model to produce a so-called “computational stain”. Additionally, the semi-supervised initialization for the lymphocyte CNN was achieved by leveraging an unsupervised convolutional autoencoder (Saltz et al., 2018). Similarly, Chuang et al. developed a DL model capable of detecting micrometastasis on annotation-free whole slide images (Chuang et al., 2021). Again, this model was based on a CNN (ResNet-50) architecture. The model was only presented WSI being annotated as either positive or negative. However, by applying the class activation mapping (CAM) method (Zhou et al., 2016), the authors could show that the network based its decision on the tumor cells. The aforementioned ResNet-50 architecture has also been used to identify axillary lymph node involvement in patients with early-stage breast cancer. Zhen et al. adapted the model to receive ultrasound and shear wave elastography images as input. Additionally, the authors included clinical information of patients in their study. By extracting the features of the CNN and combining them with the clinical status, a support vector machine was trained to predict the patient status (Zheng et al., 2020).

Additionally, DL was successfully used to identify predictive features. Koyuncu and Lu et al. showed the predictiveness of their proposed multinucleation index for p16+ oropharyngeal carcinoma. Their proposed model calculates the multinucleation index on H&E stains using two conditional Generative Adversarial Networks (cGANs). While the first neural network is used to detect multinucleation (MN) events, the second cGAN segments cancer nuclei in epithelial regions. To calculate the multinucleation index, the ratio of multinucleation events and epithelial nuclei was considered (Koyuncu et al., 2021). Furthermore, Da et al. quantified the morphological characteristics and atypia of signet ring cells (SRCC) using DL. Deep Layer Aggregation (Yu et al., 2017), a neural network-based approach, was used to segment the cells and corresponding nuclei (Da et al., 2022). The resulting quantification of morphology allows the prediction of biological behavior of SRCC.

### Applications in highly multiplexed images

Considering the availability of spatial information, the most downstream analysis that will follow the annotation of cells is automated detection and analysis of tissue patterns, which enable scientists to unravel the correlation between the high-level vision of medicine (e.g., disease diagnosis, and treatment) and the deeper levels of the underlying biological organization/mechanisms.

For instance, Schürch et al. analyzed the colorectal cancer invasive front and by calculating the composition of the k-neighborhood of each cell, higher-level features (cellular neighborhoods) were generated enabling the assignment of each cell to a primary tissue type (e.g., smooth muscle, bulk tumor). Interestingly, an association between specific cellular neighborhoods and the overall survival of colorectal cancer patients was shown (Schürch et al., 2020). Furthermore, studying cutaneous T-cell lymphoma, the authors established a spatial score based on the distances of cell types in the tissue topography, which was associated with the outcomes of pembrolizumab therapy (Phillips et al., 2021). In another study, the TME was dissected into three hierarchical levels, termed “local cell phenotypes”, “cellular neighborhoods”, and “tissue areas” (the interactions between the neighborhoods). The TME elements on these hierarchical levels were learned by a multilevel DL-based method ‘NaroNet’. Afterwards, an association between the learned TME elements and patient labels was established such that the model could perform a classification task based on the learned TMEs (Jiménez-Sánchez et al., 2022). Furthermore, a differential TME analysis was done to investigate which TMEs were mainly guiding for a specific patient label prediction. NaroNet was trained on breast cancer and endometrial carcinoma datasets and could successfully relate TMEs with patient survival risk. Furthermore, NaroNet learned to relate the TMEs with patient level labels: Copy number variation, somatic polymerase E mutations, serous-like carcinoma, and endometrioid carcinoma. Since NaroNet has the capability of suggesting interpretations on three complexity levels as described above, it stands as one of the most powerful computational pipelines that could be used to get biological insights from highly multiplexed images (Jiménez-Sánchez et al., 2022). Another association was discovered by Babaei et al. using S^3^-CIMA (supervised spatial single-cell image analysis), which is a weakly supervised convolutional neural network model. S^3^-CIMA identifies cell-cell interactions and cellular compositions and associates them with patient outcomes (Babaei et al., 2023).

Tissue-based cyclic immunofluorescence (t-CyCIF) (Lin et al., 2018) images of colorectal cancer were analyzed with spatial statistics and supervised machine learning to identify cell states and types with morphologies of known prognostic and diagnostic significance. Interestingly, the spatial analysis of the entire tumor region showed a correlation between molecular gradients and repeated transitions between histological archetypes as morphologies and tumor grades (Lin et al., 2021). Recently, Graph Neural Networks (GNNs) (Scarselli et al., 2009) were used to model the TME. For instance, a two-layer GNN was used to analyze the TME of multiplexed immunofluorescence images (IF). The analysis defined biologically meaningful compartments and predicted tumor stages. In addition, with the aid of GNNExplainer (Ying et al., 2019), the GNN identified the top features (the average expression of CD20, the FoxP3+, and epithelial cells’ interactions, and the proportion of CD4^+^ and CD8^+^ cells’ interactions) deciding the tumor stage classification (Martin et al., 2022).

Finally, Kim et al. proposed UTAG (unsupervised discovery of tissue architecture with graphs) as an unsupervised learning method to detect tissue types. Using unsupervised learning saves the need for training examples and manual annotation. UTAG was applied and validated on healthy and diseased lung tissue (Kim et al., 2022).

All the methods discussed in Image analysis are summarized in Table 2.

**TABLE 2:** The core learning approach/model used in image analysis and the corresponding clinical relevance.

Machine Learning approach/model	Data type for training the model	Clinical relevance	References
Conventional ‘simple’ images			
ResNet-101	multi-phase computed tomography images of renal tumors	Classify the renal tumors into its major subtypes	Uhm et al. (2021)
ResNet18	Microfluidic technology of different lung cancer cell lines	Classify between five distinct lung cancer cell lines and a healthy cell-line	Hashemzadeh et al. (2021)
ResNet-18 with SENet backbone	Bimodal and multimodal Ultrasound images, including ultrasound elastography images, ultrasound color Doppler and ultrasound B-mode	Predict the risk of breast malignancy	Qian et al. (2021)
Inception V3	Whole slide histological images of colorectal cancer	Classify colorectal cancer samples into cancerous and noncancerous	Yu et al. (2021)
CNN and VGG16	H&E-stained whole slide images of breast tumors	Predict breast cancer grade, estrogen receptor status, histologic subtype, PAM50 intrinsic subtype and the risk of recurrence score	Couture et al. (2018)
3D RP-Net and multi-stream Siamese subnetwork	MRI scans of rectal cancer patients	Predict the pathologic complete response (pCR) after neoadjuvant chemoradiotherapy	Jin et al. (2021)
CNN and RNN	CT scan images of metastatic colorectal cancer	Predict anti-VEGF therapy outcomes	Lu et al. (2021)
ResNet50	CT scans were used from a pilot study of intrahepatic cholangiocarcinoma patients	Predict the recurrence risk after surgery	Wakiya et al. (2022)
se-resnetxt50_32 × 4d besides a fully connected block	H&E-stained formalin-fixed primary breast tumor tissue images	H&E-ERBB2 score and was correlated with the Distant Disease-Free Survival ‘DDFS’	Bychkov et al. (2021)
RNN	Quantitative ultrasound multiparametric images of breast cancer	Predict the neo-adjuvant chemotherapy response	Taleghamar et al. (2022)
ResNet50	Ultrasound and shear wave elastography images of breast cancer	Identify axillary lymph node involvement in early-stage breast cancer	Zheng et al. (2020)
CNN	H&E stainings, 13 different tumor types	Detect tumor necrosis and tumor-infiltrating lymphocytes	Saltz et al. (2018)
Deep Layer Aggregation	Whole slide images of signet ring cell carcinoma	Analyze signet ring cells	Da et al. (2022)
cGAN	p16+ oropharyngeal carcinoma H&E stainings	Calculation of a predictive index for p16+ oropharyngeal carcinoma	Koyuncu et al. (2021)
CNN	Whole slide images of colorectal cancer	Detection of micrometastasis	Chuang et al. (2021)
Highly multiplexed images			
multilevel DL model ‘NaroNet’	Breast cancer mass cytometry images and seven-color multiplex-immunostained endometrial carcinoma images	Associate TME hierarchical levels with patient survival risk	Jiménez-Sánchez et al. (2022)
spatial statistics and supervised model	Tissue-based cyclic immunofluorescence images of colorectal cancer	Correlate molecular gradients and repeated transitions between histological archetypes as morphologies and tumor grades	Lin et al. (2021)
GNN	Colorectal cancer multiplexed immunofluorescence images	Define biologically meaningful compartments and predict tumor stages	Martin et al. (2022)
CNN (S3-CIMA)	CODEX images of colorectal cancer	Cell interactions, cell niches	Babaei et al. (2023)
GCN (STELLAR)	CODEX images of Barrett’s esophagus	Detection of tissue structure	Brbić et al. (2022)
Unsupervised Graph-based Model (UTAG)	IMC images of healthy lung and infected lung	Tissue architecture	Kim et al. (2022)

## Applications in spatial transcriptomics

Spatial transcriptomics combines tissue imaging and RNA sequencing and provides both spatial location and expression profiles of cells. The complexity and noisiness of the generated data require efficient computational tools to obtain useful biological information. To this end, DL is utilized in several spatial transcriptomics computational tools, as outlined in Table 3.

**TABLE 3:** The core learning approach/model of each spatial transcriptomics data analysis pipeline and the corresponding applications.

Method	Learning approach/model	Method highlights	References
CoSTA	Convolutional neural network	Find spatial similarities among the expression of many genes	Xu and McCord (2021)
ConST	Contrastive learning	Interpretability feature	Zong et al. (2022)
DEEPsc	Feedforward neural network	References mapping	Maseda et al. (2021)
DeepST	Transfer learning, graph neural network autoencoder, denoising autoencoder, adversarial neural network	Integrate spatial transcriptome data from several technologies or batches	Xu et al. (2022)
SpaCell	Transfer learning, convolutional neural network, feedforward neural network	Integrate spatial gene expression and image pixel data	Tan et al. (2020b)
SpaGCN	Graph convolutional network	Integrate histology, spatial location and gene expression data	Hu et al. (2021)
stLearn	Transfer learning, convolutional neural network	Integrate tissue morphology, spatial dimensionality, and the genome-wide transcriptional cellular profile	Pham et al. (2020)
Tangram	Siamese neural network, U-Net	Map snRNA-seq data to spatial data of different resolutions, ISH associated with histological and anatomical coordinates, mid-resolution Spatial Transcriptomics, and high-resolution STARmap (Wang et al., 2018) and MERFISH	Biancalani et al. (2021)
DestVI	Deep generative models (single cell Latent Variable Model ‘scLVM’, and spatial transcriptomics Latent Variable Model ‘stLVM’)	Determine the proportions of different cell types within a tissue sample and their corresponding cell states	Lopez et al. (2022)
DSTG	Graph convolutional network	Predict the composition of cell types	Song and Su (2021)
SCAN-IT	Graph convolutional network	Treats the spatial domain determination problem as an image segmentation problem, such that cells are the pixels and gene expression values of a cell are the color channels	Cang et al. (2023)
MAPLE	Graph neural network	Quantifies the effect of covariates (e.g., treatment responders vs non-responders) on tissue architecture	Allen et al. (2022)
MEFISTO	Gaussian process factor model	Factor analysis for variables with continuous covariates (e.g., spatial coordinates)	Velten et al. (2022)

Machine learning analysis can be subdivided into the following tasks and has been addressed by the different approaches: (Siegel et al., 2023): identification of spatial expression covariance patterns (CoSTA, SpaGCN, MEFISTO), (Gill et al., 2004), low dimensional representations (ConST, SpaCell), (Cone et al., 2020), cell type and state annotation (DEEPsc, DestVI), possibly accounting for low-resolution ST approaches including cell type deconvolution (DSTG, MAPLE) or tissue type annotation (SCAN-IT) and (Sun et al., 2020) integration of spatial transcriptomics and conventional pathology images (DeepST) or suspension based single-cell/single-nucleus RNA sequencing data (Tangram). Pipelines such as stLearn have been developed to cover multiple of the above tasks. Below follows a more detailed description of the above approaches.

CoSTA. A DL-based approach that utilizes convolutional neural network clustering to find spatial similarities among the expression of many genes. Furthermore, CoSTA finds spatially related expressions of genes in a context of plausible biological information. Contrary to other methods, CoSTA captures a narrow range of genes whose expression is spatially related, making it a good candidate for researchers interested in decreasing the number of related genes for further experiments (Xu and McCord, 2021).

SpaGCN. A graph convolutional network (GCN)-based approach. It is an adaptive method in analyzing various spatially resolved transcriptomics data types, such as spatial transcriptomics, STARmap, MERFISH, 10x Visium, and SLIDE-seqV2 (Stickels et al., 2021). SpaGCN is a technique that combines spatial position, gene expression, and histology to simulate the spatial dependence of gene expression in order to discover the spatial domains and domain-dense spatially variable genes SVGs. SpaGCN ensures spatial expression patterns in the SVGs it detects (Hu et al., 2021).

MEFISTO. A factor analysis approach that includes Gaussian process priors for factors over variables with continuous covariates. While these covariates are typically time points from time series data, MEFISTO has also been demonstrated on spatial covariates from spatial transcriptomics data (Velten et al., 2022).

ConST. A contrastive learning-based method that takes multi-modal data (spatial information, gene expression, and morphology) as its input and returns low-dimensional expressive embeddings as output. ConST embeddings output could be used in several downstream tasks such as cell-cell interaction, clustering, and trajectory and pseudo-time inference. Furthermore, GNNExplainer defined which spots ConST used to make its predictions. This interpretability feature of ConST nominates it to be used for more complex studies as its predictions could be explained and hence checked whether they are biologically plausible (Zong et al., 2022).

SpaCell. A cutting-edge piece of software that uses deep neural networks to combine image pixel data with thousands of spatially barcoded spots in tissue that represent different body regions. SpaCell outperforms using gene-count data alone or pixel information alone in autonomously and statistically classifying cell types and disease phases (Tan X. et al., 2020).

DEEPsc. A DL-based method that maps the spatial information on a reference atlas of single-cell RNA sequencing data. DEEPsc takes two vectors, a low-dimensional vector of a single position in the spatial reference atlas and the corresponding vector of the gene expression for a single cell. The output of DEEPsc is the likelihood that the input cell is actually at the input position (Maseda et al., 2021).

DestVI. Lopez et al. proposed DestVI, a method for deconvolving spatial transcriptomics profiles (Lopez et al., 2022). In detail, DestVI is designed to determine the proportions of different cell types within a tissue sample and identify continuous sub-states within those cell types. It is based on two different latent variable models and, thus, a probabilistic method. The input for DestVI consists of a pair of transcriptomics datasets. One from a spatial transcriptomics study and the other from a scRNA-seq study of the same tissue. The scRNA-seq data must be annotated with cell-type labels. DestVI generates output consisting of two components: first, the predicted proportion of cell types for each spot, and second, the continuous estimate of the cell state for each cell type in each spot, which reflects the average state of cells of that type in that spot.

DSTG. Song et al. developed a method for deconvoluting spatial transcriptomics data through graph-based convolutional networks (Song and Su, 2021). The model requires both real spatial transcriptomics data and pseudo spatial transcriptomics data. The pseudo-data is generated from single-cell RNA sequencing data of the same tissue. Furthermore, a link graph between both datasets is learned to connect similar spots, also used as input for the method. DSTG is used to predict the composition of cell types in the real spatial transcriptomics data. This goal is achieved by not only using the features of each spot but also the known composition of spots in the real spatial transcriptomics data encoded in the graph.

MAPLE. (Multi-sAmple sPatiaL transcriptomics modEl) is a modeling framework for multi-sample spatial transcriptomics data leveraging machine learning and Bayesian statistical modeling, which was proposed by Allen et al. The method aims to simultaneously detect cell spot sub-populations on multiple samples while accounting for uncertainty. First, spatially aware gene expression features are extracted using scGNN (Allen et al., 2022).

Using the extracted features, spatially informed cell subpopulations are detected in each sample using a Bayesian multivariate finite mixture model. The priors are selected to induce a correlation between cell spots within each sample. Lastly, MAPLE quantifies the effect of covariates (e.g., treatment responders vs non-responders) on tissue architecture by using a multinomial logistic regression model for the spot-level mixture component probabilities.

SCAN-IT. Proposed by Cang et al. is a GCN-based method for tissue identification in spatial transcriptomics data (Cang et al, 2021). To solve this problem, they state spatial domain identification as an instance of image segmentation. Each cell corresponds to a pixel with its gene expressions representing color channels. In the first step, a graph representation of the spatial spots is generated by using the alpha complex. Following that, multiple GCNs are used as encoders. Using the resulting embeddings, consensus clustering is performed to obtain the segmented tissue domains.

DeepST. A DL-based method that utilizes a pretrained neural network that is fed with morphological images to extract feature vectors from them. Afterwards, it takes gene expression and spatial data, and integrates them with the extracted feature vectors forming a spatial augmented gene expression matrix. Furthermore, DeepST exploits a GNN autoencoder besides a denoising autoencoder to create a latent representation of the augmented spatial transcriptome data. Moreover, DeepST uses domain adversarial neural networks to integrate spatial transcriptome data from several technologies or batches (Xu et al., 2022).

Tangram. An approach for matching single-cell and single-nucleus RNA sequencing data to a wide difference of spatial information gathered from the same area, such as histology images, MERFISH, STARmap, smFISH (Femino et al., 1998), and Visium. Tangram is a deep-learning model that integrates histological, morphological, and *in vivo* findings with single-cell and single-nucleus RNA sequencing data offering high-sensitive spatial measurements and an atlas with high resolution. Tangram has been developed and expanded to be employed to different organs and tissue diseases (Biancalani et al., 2021).

stLearn is a pioneering interdisciplinary analysis method that uses three different data categories (tissue morphology, spatial dimensionality, and the genome-wide transcriptional cellular profile). stLearn allows scientists to use spatial transcriptomics data as effectively as possible. In a morphologically intact tissue sample, stLearn starts with cell type identification, rebuilds cell type evolution within the tissue, then explores tissue locations with strong cell-cell interconnections (Pham et al., 2020).

In this section, we covered the DL-methods for spatial transcriptomics data and the kind of information that could be obtained from each pipeline. Although DL methods suffer from expensive computations and might not offer biological interpretations (Heydari and Sindi, 2023), this gap could be covered by continuous efforts to come up with interpretable models (Weld and Bansal, 2018; Elmarakeby et al., 2021).

## Discussion and conclusion

Machine learning, particularly DL, is rapidly expanding and promising in medical imaging applications, and it is anticipated that DL will become incorporated into the standard techniques of medical imaging in the next decade (Suzuki, 2017). DL algorithms can outperform the existing clinical procedures by extracting supplementary histological information of solid tumors that are not derived from routine analysis. As a result, deep neural networks have proven their ability to fully influence clinical decision-making in solid tumor treatment (Echle et al., 2021).

Recent DL developments have illuminated medical image analysis by facilitating image registration and identifying anatomical and cellular structures and/or textural patterns in images dependent only on data. Tissue segmentation (Zhang et al., 2015), image fusion (Suk et al., 2014), image annotation (Shin et al., 2016), microscopic imaging analysis (Cireşan et al., 2013), computer-aided disease detection or prediction (Suk et al., 2015), and lesion/landmark identification (Pereira et al., 2016) were further successful applications of DL (Shen et al., 2017). The Food and Drug Administration has so far authorized several DL techniques to be applied in radiology and pathology. Advanced DL applications surpass the existing pathologists’ routine reporting (Echle et al., 2021).

Even though certain DL-based diagnostic frameworks have already acquired official permission to be applied in conventional clinical processes (Topol, 2019), independent verification and comprehensive review of these innovations are still in the early stages (Beam and Kohane, 2018). Thus, to confirm the a model validity in clinical practice (Uhm et al., 2021), and to improve efficacy, additional experiments with greater numbers of samples are required. Two intriguing future approaches for DL-based single-cell optical image investigations are developing specialized DL algorithms with biometric translation capabilities and establishing open-source databases of single-cell optical images (Qian et al., 2021).

In this review, we discussed how successful DL is in enhancing each step in highly multiplexed imaging, from image segmentation to cell type annotation and even biological interpretations of the generated images. Furthermore, we outlined that on the one hand, the importance of including DL in the clinical routine as its combination with simple images will help physicians to generate better disease diagnoses, prognoses, and therapy outcome predictions. On the other hand, for researchers, the upgrade from DL/simple imaging to DL/highly multiplexed imaging is advised, as the latter can provide hypotheses on and insights into the mechanisms of disease initiation and progression, which, if experimentally validated, will ultimately improve medical care for the patients.
