# Corrigendum: A review on deep learning applications in highly multiplexed tissue imaging data analysis

**DOI:** 10.3389/fbinf.2023.1287407

**Published:** 2023-09-13

**Authors:** Mohammed Zidane, Ahmad Makky, Matthias Bruhns, Alexander Rochwarger, Sepideh Babaei, Manfred Claassen, Christian M. Schürch

**Affiliations:** ^1^ Department of Pathology and Neuropathology, University Hospital and Comprehensive Cancer Center Tübingen, Tübingen, Germany; ^2^ Department of Internal Medicine I, University Hospital Tübingen, Tübingen, Germany; ^3^ Department of Computer Science, University of Tübingen, Tübingen, Germany

**Keywords:** artificial intelligence, deep learning, highly multiplexed tissue imaging, spatial transcriptomics, cancer, biomarker, prediction, review

In the published article, there was an error. “The abstract is duplicated.”

A correction has been made to **Abstract**.

This sentence previously stated:

“Since its introduction into the field of oncology, deep learning (DL) has impacted clinical discoveries and biomarker predictions. DL-driven discoveries and predictions in oncology are based on a variety of biological data such as genomics, proteomics, and imaging data. DL-based computational frameworks can predict genetic variant effects on gene expression, as well as protein structures based on amino acid sequences. Furthermore, DL algorithms can capture valuable mechanistic biological information from several spatial “omics” technologies, such as spatial transcriptomics and spatial proteomics. Here, we review the impact that the combination of artificial intelligence (AI) with spatial omics technologies has had on oncology, focusing on DL and its applications in biomedical image analysis, encompassing cell segmentation, cell phenotype identification, cancer prognostication, and therapy prediction. We highlight the advantages of using highly multiplexed images (spatial proteomics data) compared to single-stained, conventional histopathological (“simple”) images, as the former can provide deep mechanistic insights that cannot be obtained by the latter, even with the aid of explainable AI. Furthermore, we provide the reader with the advantages/disadvantages of DL-based pipelines used in preprocessing highly multiplexed images (cell segmentation, cell type annotation). Therefore, this review also guides the reader to choose the DLbased pipeline that best fits their data. In conclusion, DL continues to be established as an essential tool in discovering novel biological mechanisms when combined with technologies such as highly multiplexed tissue imaging data. In balance with conventional medical data, its role in clinical routine will become more important, supporting diagnosis and prognosis in oncology, enhancing clinical decision-making, and improving the quality of care for patients. Since its introduction into the field of oncology, deep learning (DL) has impacted clinical discoveries and biomarker predictions. DL-driven discoveries and predictions in oncology are based on a variety of biological data such as genomics, proteomics, and imaging data. DL-based computational frameworks can predict genetic variant effects on gene expression, as well as protein structures based on amino acid sequences. Furthermore, DL algorithms can capture valuable mechanistic biological information from several spatial “omics” technologies, such as spatial transcriptomics and spatial proteomics. Here, we review the impact that the combination of artificial intelligence (AI) with spatial omics technologies has had on oncology, focusing on DL and its applications in biomedical image analysis, encompassing cell segmentation, cell phenotype identification, cancer prognostication, and therapy prediction. We highlight the advantages of using highly multiplexed images (spatial proteomics data) compared to single-stained, conventional histopathological (“simple”) images, as the former can provide deep mechanistic insights that cannot be obtained by the latter, even with the aid of explainable AI. Furthermore, we provide the reader with the advantages/ disadvantages of the DL-based pipelines used in preprocessing the highly multiplexed images (cell segmentation, cell type annotation). Therefore, this review also guides the reader to choose the DL-based pipeline that best fits their data. In conclusion, DL continues to be established as an essential tool in discovering novel biological mechanisms when combined with technologies such as highly multiplexed tissue imaging data. In balance with conventional medical data, its role in clinical routine will become more important, supporting diagnosis and prognosis in oncology, enhancing clinical decision-making, and improving the quality of care for patients.”

The corrected sentence appears below:

“Since its introduction into the field of oncology, deep learning (DL) has impacted clinical discoveries and biomarker predictions. DL-driven discoveries and predictions in oncology are based on a variety of biological data such as genomics, proteomics, and imaging data. DL-based computational frameworks can predict genetic variant effects on gene expression, as well as protein structures based on amino acid sequences. Furthermore, DL algorithms can capture valuable mechanistic biological information from several spatial “omics” technologies, such as spatial transcriptomics and spatial proteomics. Here, we review the impact that the combination of artificial intelligence (AI) with spatial omics technologies has had on oncology, focusing on DL and its applications in biomedical image analysis, encompassing cell segmentation, cell phenotype identification, cancer prognostication, and therapy prediction. We highlight the advantages of using highly multiplexed images (spatial proteomics data) compared to single-stained, conventional histopathological (“simple”) images, as the former can provide deep mechanistic insights that cannot be obtained by the latter, even with the aid of explainable AI. Furthermore, we provide the reader with the advantages/disadvantages of DL-based pipelines used in preprocessing highly multiplexed images (cell segmentation, cell type annotation). Therefore, this review also guides the reader to choose the DL-based pipeline that best fits their data. In conclusion, DL continues to be established as an essential tool in discovering novel biological mechanisms when combined with technologies such as highly multiplexed tissue imaging data. In balance with conventional medical data, its role in clinical routine will become more important, supporting diagnosis and prognosis in oncology, enhancing clinical decision-making, and improving the quality of care for patients.”

In the published article, there was an error. “the word ‘recently’ is repeated.”

A correction has been made to **Applications in highly multiplexed images**, [paragraph 3].

This sentence previously stated:

“Recently, Graph Neural Networks (GNNs) (Scarselli et al., 2009) were recently used to model the TME.”

The corrected sentence appears below:

“Recently, Graph Neural Networks (GNNs) (Scarselli et al., 2009) were used to model the TME.”

In the published article, there was an error. “the term ‘convolutional neural network’ is repeated”.

A correction has been made to **Applications in conventional medical (“simple”) images**, [paragraph 5].

This sentence previously stated:

“The DL-based framework consists of two neural networks: a convolutional neural network: a convolutional neural network CNN (pre-trained GoogleNet on ImageNet), which was trained on the CT scans to extract the important features from lesions of different organs, and a recurrent neural network RNN which learned the changes happening in these lesions across multiple time points.”

The corrected sentence appears below:

“The DL-based framework consists of two neural networks: a convolutional neural network CNN (pre-trained GoogleNet on ImageNet), which was trained on the CT scans to extract the important features from lesions of different organs, and a recurrent neural network RNN which learned the changes happening in these lesions across multiple time points.”

In the published article, there was an error. “two references are not written as a hyperlink; it is just a number that you cannot click on”.

A correction has been made to reference (148) in **Table 1** is not a hyperlink and reference (171) in **Table 3**. Both references are written as numbers not as hyperlinks.

This sentence previously stated:

“Could be combined with TrackMate (148) for cell tracking in **Table 1**.”

“Map snRNA-seq data to spatial data of different resolutions, ISH associated with histological and anatomical coordinates, midresolution Spatial Transcriptomics, and high-resolution STARmap (171) and MERFISH in **Table 3**.”

The corrected sentence appears below:

“They should be hyperlinks.”

The authors apologize for these errors and state that this does not change the scientific conclusions of the article in any way. The original article has been updated.

